# Trabectedin triggers direct and NK-mediated cytotoxicity in multiple myeloma

**DOI:** 10.1186/s13045-019-0714-9

**Published:** 2019-03-21

**Authors:** Maria Cucè, Maria Eugenia Gallo Cantafio, Maria Anna Siciliano, Caterina Riillo, Daniele Caracciolo, Francesca Scionti, Nicoletta Staropoli, Valeria Zuccalà, Lorenza Maltese, Anna Di Vito, Katia Grillone, Vito Barbieri, Mariamena Arbitrio, Maria Teresa Di Martino, Marco Rossi, Nicola Amodio, Pierosandro Tagliaferri, Pierfrancesco Tassone, Cirino Botta

**Affiliations:** 1 Department of Experimental and Clinical Medicine, Magna Graecia University, Salvatore Venuta University Campus, Viale Europa, 88100 Catanzaro, Italy; 2 Pathology Unit, AO Pugliese-Ciaccio, Catanzaro, Italy; 3 Medical and Translational Oncology Units, AOU Mater Domini, Catanzaro, Italy; 4 Institute of Neurological Sciences, UOS of Pharmacology, Catanzaro, Italy; 5 Sbarro Institute for Cancer Research and Molecular Medicine, Center for Biotechnology, College of Science and Technology, Temple University, Philadelphia, PA USA

**Keywords:** Myeloma, 3D-models, Natural killer, Micro-RNAs, Trabectedin

## Abstract

**Background:**

Genomic instability is a feature of multiple myeloma (MM), and impairment in DNA damaging response (DDR) has an established role in disease pathobiology. Indeed, a deregulation of DNA repair pathways may contribute to genomic instability, to the establishment of drug resistance to genotoxic agents, and to the escape from immune surveillance. On these bases, we evaluated the role of different DDR pathways in MM and investigated, for the first time, the direct and immune-mediated anti-MM activity of the nucleotide excision repair (NER)-dependent agent trabectedin.

**Methods:**

Gene-expression profiling (GEP) was carried out with HTA2.0 Affymetrix array. Evaluation of apoptosis, cell cycle, and changes in cytokine production and release have been performed in 2D and 3D Matrigel-spheroid models through flow cytometry on MM cell lines and patients-derived primary MM cells exposed to increasing nanomolar concentrations of trabectedin. DNA-damage response has been evaluated through Western blot, immunofluorescence, and DNA fragmentation assay. Trabectedin-induced activation of NK has been assessed by CD107a degranulation. miRNAs quantification has been done through RT-PCR.

**Results:**

By comparing GEP meta-analysis of normal and MM plasma cells (PCs), we observed an enrichment in DNA NER genes in poor prognosis MM. Trabectedin triggered apoptosis in primary MM cells and MM cell lines in both 2D and 3D in vitro assays. Moreover, trabectedin induced DDR activation, cellular stress with ROS production, and cell cycle arrest. Additionally, a significant reduction of MCP1 cytokine and VEGF-A in U266-monocytes co-cultures was observed, confirming the impairment of MM-promoting *milieu*. Drug-induced cell stress in MM cells led to upregulation of NK activating receptors ligands (i.e., NKG2D), which translated into increased NK activation and degranulation. Mechanistically, this effect was linked to trabectedin-induced inhibition of NKG2D-ligands negative regulators IRF4 and IKZF1, as well as to miR-17 family downregulation in MM cells.

**Conclusions:**

Taken together, our findings indicate a pleiotropic activity of NER-targeting agent trabectedin, which appears a promising candidate for novel anti-MM therapeutic strategies.

**Electronic supplementary material:**

The online version of this article (10.1186/s13045-019-0714-9) contains supplementary material, which is available to authorized users.

## Background

Multiple myeloma (MM) is still an incurable hematologic malignancy characterized by clonal proliferation of malignant plasma cells (PCs) within the bone marrow (BM). Current MM therapy includes triple- or double-drug combination, based on proteasome inhibitors (PIs) and/or immune-modulatory drugs (IMiDs) *plus* dexamethasone, with or without chemotherapeutic agents [1]. Autologous stem cell transplant is reserved to selected patients as consolidation following induction treatment. However, despite recent advancements that significantly improved clinical outcome, patients invariably progress to drug resistance.

DNA repair mechanisms have a crucial role for the maintenance of the genome integrity, and their activation is fine tuned to resolve specific DNA damages. Currently, at least seven DNA repair active systems have been described in MM as protection from different DNA lesions [2]. Specifically, base excision repair (BER), nucleotide excision repair (NER), and mismatch repair (MMR) pathways are involved in the repair of single-strand DNA damages; homologous recombination (HR), classical non-homologous end joining (c-NHEJ), and alternative NHEJ (a-NHEJ) pathways are conversely involved in double-strand breaks (DSBs), while Fanconi anemia pathway (together with NER and HR) is involved in the repair of interstrand crosslinks [2, 3]. Dysregulation of these systems has been found to promote tumor progression, cell survival, and development of drug resistance [2–4]. Furthermore, activation of DNA damage response (DDR) has been involved in the upregulation of ligands for activating receptors of natural killer (NK) lymphocytes. Indeed, besides participating in cell cycle control and induction of apoptosis, DDR works as a sensor for cellular stress or transformation, inducing recognition by the immune system [5, 6].

Genomic instability is a major hallmark of MM and most of the drugs currently used in the treatment of MM have direct genotoxic activity (i.e., melphalan, doxorubicin, cyclophosphamide) or interfere with the DNA repair machinery (PIs or IMiDs) [2]. Accordingly, these drugs have been reported to trigger the expression of DNAM-1 and NKG2D ligands on MM cells and to induce NK cells activation [7, 8].

Herein, the expression and prognostic relevance of genes of DNA repair pathways in MM has been investigated. Since overexpression of NER pathway has been found, evaluation of the direct and immune-mediated anti-MM activity of the NER-targeting agent trabectedin in 2D and 3D experimental models of MM has been performed.

## Methods

### Cell lines, MM primary cells, and drugs

Multiple myeloma cell lines were cultured at 37 °C with 5% CO_2_. AMO-1, U266, and NCI-H929, SKMM1 were purchased from DSMZ (Braunschweig, Germany). AMO-BZB and AMO-CFZ were kindly provided by Dr. Christoph Driessen (Eberhand Karls University, Tübingen Germany), MM1S and RPMI-8226 were purchased from ATCC (Manassas, VA, USA), and OPM2 and RPMI-8226 DOX40 were kindly provided by Dr. K.C. Anderson (Dana-Farber Cancer Institute, Harvard Medical School, Boston, MA, USA). All these cells were cultured in RPMI-1640 medium (Gibco, Life Technologies) supplemented with 10% heat-inactivated fetal bovine serum (FBS) and 100 U/mL penicillin, and 100 μg/mL streptomycin (GIBCO; Thermo Fischer, Carlsbad, CA). U266 and U266 LR7 (kindly provided by Dr. A. Pandiella, Instituto de Biología Molecular y Celular del Cáncer, CSIC-Universidad de Salamanca, Salamanca, Spain) were cultured in RPMI-1640 with 20% FBS and 100 U/mL penicillin, and 100 μg/mL streptomycin. JJN3 were purchased from DSMZ and were cultured in Dulbecco’s modified Eagle medium supplemented with 20% FBS and 100 U/mL penicillin, and 100 μg/mL streptomycin.

NK-92 CI were obtained from NantKwest (Dr. Kerry S. Campbell) and cultured in alpha-MEM medium with ribonucleosides and deoxyribonucleosides (Gibco, Thermo Scientific) supplemented with 10% horse serum, 10% FBS, 0.2 mM myo-inositol (Sigma), 2 mM l-glutamine, 0.1 mM β-mercaptoethanol (Sigma), 0.002 mM folic acid (Fisher Scientific), 1x NEAA (Gibco, Thermo Scientific), 1 mM Na pyruvate (Gibco, Thermo Scientific), 100 U/mL penicillin, and 100 μg/mL streptomycin (Life Technologies) and 100 IU/mL of recombinant human IL-2 (IL-2 improved sequence, Miltenyi).

Primary MM cells were immune-magnetically sorted by using CD138 MicroBeads (MACS, Miltenyi, according to producers’ guidelines) from leftover samples of three MM patients’ bone marrow aspirates after all diagnostic procedures. All patients had provided the informed consent according to institutional bioethical standards, and all the samples have been anonymized before use (institutional approval n. 120/2015, within the project Innovative immunotherapeutic treatments of human cancer, MultiUnit—Multi Unit Regional n.16695).

Human monocytes were obtained by immune magnetical separation from healthy donor peripheral blood mononuclear cells (PBMCs) after Ficoll-Paque density-gradient separation. Specifically, BD IMag™ anti-human CD14 magnetic particles were used for positive selection of CD14^+^ monocytes according to producers’ guidelines.

Ascorbic acid, as antioxidant agent, was purchased from Sigma-Aldrich (Saint Louis, USA).

Trabectedin (PharmaMar, Madrid, Spain) was reconstituted in DMSO.

### Virus generation and transduction of MM cells

MM cells stably expressing miR-17-92 cluster have been gently provided by Dr. E. Morelli. These cells have been obtained by using a PMIRH17-92PA-1 lenti-vector (System Biosciences, Palo Alto, CA, USA) through a methodology already described elsewhere [9, 10]. Briefly, packaging of the miR-17-92 cluster constructs in pseudoviral particles was performed in 293Ta cells using the Lenti-Pac FIV Expression Packaging Kit (FPKLvTR-20), according to the manufacturer’s instructions (Genecopoeia, Rockville, MD, USA). After transfection of 293 T cells, supernatants containing miR-17-92 lentivirus were collected at 8-h intervals, filtered, and used for two rounds of transduction of U266 cells (1 × 10^6^) in the presence of 8 mg/mL of polybrene (Sigma-Aldrich). Two days after transduction, selection with 1 μg/ml puromycin for 3 days was performed to achieve almost 100% transduced cells. Empty lentivirus transduced cells were used as a control for the experiments.

### Apoptosis evaluation

MM cells (2 × 10^5^) were treated with dose escalation of trabectedin (0-0.1-0.25-0.5-1-2.5 nM) and analyzed for apoptosis after 24, 48, and 72 h through Annexin V/7-AAD flow cytometry assay (Becton Dickinson). All experiments have been performed at least three times. Primary MM cells were exposed to 2.5 nM of Trabectedin and apoptosis was evaluated after 24 h. Apoptosis was further investigated at molecular level, analyzing the cleaved/total levels of caspase 3 and PARP by Western blot.

### Cell cycle analysis

U266 and MM1S (1X10^6^) were cultured in the presence of trabectedin (1 nM and 0.1 nM, respectively) in 6-wells plate for 48 h. Cells were then collected and washed twice with PBS 1X. Subsequently, 1 mL of 70% ice cold ethanol for each sample was added. Cells were stored at − 20 °C until used and then centrifuged and washed twice with PBS 1X. Cells were then resuspended in 1 mL of PI staining solution (100 μg/mL of ribonuclease A, 50 μg/mL of propidium iodide, and 0.01% of NP-40) and incubated for 1 h at room temperature, in the dark. Analysis was performed with flow cytometer and repeated three times.

### 3D model

We established a 3D in vitro model of MM cell lines alone or in the presence of human monocytes using Matrigel® matrix (Corning). Briefly, 1 × 10^5^ MM cells (U266, OPM2, MM1S) alone or in co-culture with 0.5 × 10^5^ CD14+ monocytes (2:1 MM/monocytes ratio) were resuspended in ice-cold matrigel and a matrigel drop of 35 μL was placed in 24-wells plate coated with a sterile parafilm dish to form a Matrigel-spheroid. After 30 min of incubation at 37 °C, 500 μL of medium with different concentrations of trabectedin was added to each well and the spheroids were incubated for 72 h. Matrigel-spheroids were then resuspended in Dispase (Sigma-Aldrich) and the recovered cells were stained with annexin-V/ 7AAD for analyzing apoptosis induction by flow-cytometry. Supernatants were collected to analyze cytokines expression. Alternatively, Matrigel-spheroids were stored for immunohistochemistry evaluation. All experiments have been performed at least three times.

### Immunohistochemistry

Matrigel-spheroids of either tumor cells alone or co-cultured with CD14^+^ monocytes isolated from healthy donors were fixed in 0.3% glutaraldehyde, then in 4.21% formaldehyde, and subsequently paraffin-embedded. Serial section of 4-μm-thick were cut and mounted on acid-cleaned glass slides, which were dewaxed with xylene, and processed for hematoxylin-eosin staining and immunohistochemistry.

Slides were incubated overnight at 4 °C with anti-g-H2ax monoclonal antibody (Cell Signaling) and anti-cleaved caspase 3 (Santa Cruz Technologies) primary antibodies, washed with PBS three times and incubated with appropriate chromogen-conjugated secondary antibody for 1 h at room temperature. After washings using PBS, samples were observed by an optical microscope and images were acquired.

### Single-cell gel electrophoresis (Comet) assay

Comet assay (Trevigen) was performed according to manufacturer’s instructions. Briefly, cells were harvested (1 × 10^5^ cells per pellet), mixed with 200 mL low-melting agarose, and layered onto agarose-coated glass slides. The slides were immersed in lysis solution, and then placed into a horizontal electrophoresis apparatus filled with fresh alkaline or neutral electrophoresis buffer. After electrophoresis (30 min at 1 V/cm tank length), air-dried and neutralized slides were stained with Dapi and kept in a moist chamber in the dark at 4 °C. Images were acquired at × 63 oil immersion with an SP2 Leica Zeiss confocal laser-scanning microscope.

### Mitochondrial membrane potential and ROS/superoxide analysis

MM cells (5 × 10^5^) were seeded in 12-wells plate and were incubated for 24 h, untreated or treated with sub-lethal doses of trabectedin (depending on cell line), in the presence or absence of ascorbic acid (25 μM), as antioxidant agent. Trabectedin-induced changes in the production of mitochondrial membrane potential (MMP) and radical oxygen species (ROS) were evaluated by MitoScreen assay (Becton Dickinson) and Total ROS/Superoxides Detection kit (ENZO Life Sciences) respectively, by flow cytometry according to producer’s guidelines.

### Flow cytometry and degranulation assay

The expression of the NKG2D and DNAM-1 ligands on different MM cells was evaluated, after 48 h of culture in the presence of trabectedin, by using fluorochrome-conjugated antibodies against MIC-A/B (Becton Dickinson), ULBP 1 (R&D Systems), ULBP 2-5-6 (R&D Systems), PVR (R&D Systems), and NECTIN-2 (Becton Dickinson) according to producer’s guidelines.

NK cell degranulation was evaluated using the CD107a staining. Specifically, trabectedin-treated MM cell lines were washed twice in complete medium and incubated with NK-92 CI cell line at effector/target (E:T) ratio of 1:1, in a U-bottom 96-well plate in complete medium at 37 °C and 5% CO_2_ in the presence of anti-CD107a/PE (Becton Dickinson) for 2 h. Cells were then stained with anti-CD3/PcP and anti-CD56/APC to identify NK cell population. NK cells positive for CD107a were considered as degranulating/activated cells able to induce cytotoxicity.

All experiments were acquired by an ATTUNE Nxt (Thermo Scientific) flow cytometer. For each sample, at least 1 × 10^4^ events in the gate of interest were acquired.

### RNA extraction and quantitative real-time-PCR

Total RNA from MM cells was prepared with TRIzol® Reagent (Life Technologies) according to manufacturer’s instructions. The integrity and quantity of total RNA was assessed using the NanoDrop Spectrophotometer (Thermo Scientific). The single-tube TaqMan miRNA assay (Life Technologies) was used to detect and quantify mature miR-17, miR-18a, miR-19a, miR-19b, miR-20a, miR-92a, performing a real-time polymerase chain reaction (RT-PCR) using TaqMan®Fast Universal PCR Master Mix on a ViiA7 RT reader (Life Technologies). MiRNAs expression was normalized on the RNU44 snoRNA (Life Technologies). Comparative RT–PCR was performed in triplicate, including no-template controls. Relative expression was calculated by using the ∆∆-cycle threshold (CT) method [11].

### Gene-expression profiling

U226 MM cells (3 × 10^6^), obtained from two different experiments, were treated with PBS or 2.5 μM of trabectedin for 24 h. Gene expression profiling was performed as described elsewhere [12]. Briefly, total RNA (tRNA) was extracted through column purification with RNeasy kit (Qiagen, Hilden, Germany). A total of 300 ng RNA was used as starting material for preparing the hybridization target by using the Ambion® WT Expression Kit (Ambion, Life Technologies). The integrity, quality, and quantity of tRNA were assessed by the Agilent Bioanalyzer 2100 (Agilent Technologies, Santa Clara, CA) and NanoDrop 1000 Spectrophotometer (Thermo Scientific, Wilmington, DE). The amplification of cRNA, the cleanup, and the fragmentation were performed according to the Affymetrix’s procedures. Microarray data were generated by Human transcriptom array 2.0 ST (Affymetrix Inc., Santa Clara, Ca). Arrays were scanned with an Affymetrix GeneChip Scanner 3000. Raw data produced by the Affymetrix Platform (i.e., CEL files) were processed and RMA normalized using Affymetrix Expression Console (EC). Data set has been deposited under the GEO accession number GSE128020.

### Gene set enrichment analysis and gene ontology

We used the gene set enrichment analysis (GSEA) [13] tool to enrich the target pathways with statistically significant differences between trabectedin treated versus untreated cells. Indeed, given a specific gene expression profile sorted by the expression ratio between the two conditions, the target pathway is considered significantly enriched if members are enriched in the top (up-regulated) or bottom (down-regulated) region of the profile. GSEA software then calculates an enrichment score (ES) by using a Kolmogorov-Smirnov test, to measure the degree to which the pathway is enriched in the top-ranked or bottom-ranked region of the profile. Next, ES is normalized (NES) according to the number of genes belonging to the pathways, in order to make comparable pathways with different size. Of note, this process takes into account the contribution of all genes included into the analysis, including those with minimum fold change. GSEA analysis parameters have been set as follows: number of permutations: 1000; permutation type: gene_set; metric for ranking genes: log2 ratio of Classes; size of genesets: 25–500 genes; gene sets evaluated: Hallmark gene sets and C2 curated gene sets from MSigDB.

Additionally, genes upregulated or downregulated with a fold change of at least 1.5 where analyzed with ClueGO, a Cytoscape plug in app that visualizes non-redundant biological terms for large clusters of gene sets in a functionally grouped network [14, 15].

### Western blot

Proteins were extracted from MM cells after lysing in NP40 CellLysis Buffer (Novex) containing a cocktail of protease and phosphatase inhibitors (Thermo Scientific, Waltham, MA). Whole cells lysates (20–30 μg/well) were loaded and separated on 4–12% NovexBis–Tris SDS–acrylamide gels or 3–8% Tris-Acetate Protein Gels (Gibco, Life Technologies). Proteins were then transferred on nitrocellulose membranes by Trans-Blot Turbo Transfer Starter System (Bio-Rad, Berkeley, CA). Subsequently, membranes were blotted with the following primary antibodies: anti-P21, anti-BCL2, anti-RAD51, anti-MCL1, anti-PRO-CASP3, anti-C-CASP3, anti-CDK6, anti-cyclinD1, anti-E2F1, anti-IKZF1, anti-XRCC1, anti-RPA32 (Cell Signaling) and anti-cPARP, anti-PARP, anti-BCL2, anti-IRF4, anti-PTEN, anti-XPF, anti-DDB2, anti-ERCC1, anti-actin, anti-vinculin, and GAPDH (Santa Cruz). To study major signaling checkpoints in response to active DNA damage, a specific commercial kit from Cell Signaling (DNA Damage Antibody Sampler Kit #9947) has been used, as already described in previous work by us and others [16–18]. The kit includes the following primary antibodies: anti-phospho (p)P53 (Ser15), anti-gammaH2AX (Ser139), anti-pATM (Ser1981), anti-pATR (Ser428), anti-pCHEK1 (Ser345), anti-pCHEK2 (Thr68), and anti-pBRCA1 (Ser1524). All these forms are virtually absent in normal conditions and are activated in response to DNA damage to induce an attempt to DNA repair and block cell cycle progression (through, for example, p21). GAPDH expression has been used as a protein loading control for this kit. Blots were then incubated with goat anti-mouse or goat anti-rabbit HRP-conjugated antibodies (Santa Cruz Biotechnology); immunoreactive bands were detected by use of enhanced chemiluminescence (ECL) method, acquired through the C-DIGIT scanner (LI-COR) and quantified by Image Studio Lite 5.0 (LI-COR).

### Immunostaining for confocal microscopy

Trabectedin-treated and control MM cells were seeded onto glass coverslips and cytospin for 5 min at 800 rpm was performed. Cells were then washed in PBS, fixed in 4% paraformaldehyde for 12 min, washed three times with PBS, followed by permeabilization with 0.01% Triton-X for 15 min, and again washed in PBS containing 0.5% BSA. Cells were then incubated with anti-g-H2ax monoclonal antibody (cell signaling) overnight at 4 °C, washed with PBS three times, and incubated with Alexa-fluor 488-conjugated secondary antibody (Molecular Probes, Life Technologies, NY) for 1 h at room temperature. Glass coverslips were then washed three times with PBS and mounted with Vecta-Shield mounting media containing DAPI. Samples were visualized and images captured using a Leica microscope. Images were acquired at × 63 oil immersion with an SP2 Leica Zeiss confocal laser-scanning microscope.

### Cytokines analysis

A panel of different cytokines including IL1b, IL4, IL6, IL8, IL23, TNF, IFNg, G-CSF, IP10, MCP1, IL10, and VEGF were detected in the supernatants of CD14^+^ cells alone obtained by PBMCs of healthy donors and co-cultured with MM cells in 3D Matrigel-spheroids, in the presence or absence of Trabectedin, using BD CBA Human Soluble Protein Flex Set system (Becton Dickinson, Heidelberg, Germany). Samples from three different experiments were analyzed with an Attune Nxt Thermo Scientific flow cytometer.

### Tube assay formation

A drop of 50 μL of Matrigel (CORNING) were used to coat 96-wells plates and allowed to polymerize at 37 °C for 30 min. Then, 15 × 10^3^ HUVECs were seeded in each well and then 50 μL of conditioned medium from trabectedin-treated cells were added. After 1-h incubation at 37 °C, at least pictures of three representative fields per well were taken using phase contrast microscopy. The tubulogenic potential was quantified by estimating the total tube length and the number of nodal branchpoints (a single pixel connected to three or more pixels), through the “Pipeline 1.4” tool [19] (https://sourceforge.net/projects/pipelinetfaanalysis/). All experiments have been performed at least three times.

### Gene expression datasets analysis

Datasets of gene expression profiling of MM were retrieved from GEO database (Table 1) or from the MMRF researcher gateway portal (https://research.themmrf.org). The GSE47552 dataset includes data from 5 healthy donors (HD), 20 patients with MGUS, 33 high-risk sMM, and 41 MM; the GSE39754 includes results from 6 HD and 170 MM; the GSE6477 includes gene expression profiles of 22 MGUS, 24 sMM, 69 newly diagnosed MM, 32 relapsed MM, and 15 healthy subjects; and the GSE13591 dataset contains the gene expression profiles of immunomagnetically purified CD138^+^ plasma cells obtained from 5 HD, 11 MGUS, 133 MM, and 9 plasma cell leukemia at diagnosis.

**Table 1:** Datasets of gene expression profiling of MM retrieved from GEO database

Database	GEO	Platform	Institute	Organism	Summary
1	GSE47552	Affymetrix Human Gene 1.0 ST Array (GPL6244)	Centro de Investigación del Cáncer de Salamanca	*Homo sapiens*	Analysis of plasma cells from patients with monoclonal gammopathy of undetermined significance (MGUS) (*n* = 20), smoldering multiple myeloma (sMM) (*n* = 33), symptomatic MM (*n* = 41), and healthy donors (*n* = 5).
2	GSE39754	Affymetrix Human Exon 1.0ST Array(GPL5175)	Dana-Farber Cancer Institute	*Homo sapiens*	Gene expression microarray datasets from CD138 purified plasma cells isolated from 170 patients with newly diagnosed MM and 6 healthy subjects. All patients received triple drug regime—Vincristine, Adriamycin, and Dexamethasone (VAD)—as induction therapy followed by autologous stem cell transplant (ASCT) as a maintenance therapy.
3	GSE6477	Affymetrix Human Genome U133A Array (GPL96)	Mayo Clinic	*Homo sapiens*	Gene expression profile of CD138 purified plasma cells from 22 MGUS, 24 sMM, 69 newly diagnosed MM, 32 relapsed MM, and 15 healthy subjects. Each sample has been further characterized by FISH for the identification of hyperdiploidy.
4	GSE13591	Affymetrix Human Genome U133A Array (GPL96)	University of Milan—Fondazione IRCCS Ospedale Maggiore Policlinico	*Homo sapiens*	This series of microarray experiments contains the gene expression profiles of immunomagnetically purified CD138^+^ plasma cells obtained from 5 normal donors, 11 MGUS, 133 MM, and 9 plasma cell leukemia at diagnosis

The CoMMpass (Relating Clinical Outcomes in MM to Personal Assessment of Genetic Profile) Trial (NCT0145429), a longitudinal study in MM, relating clinical outcomes to genomic and immune-phenotypic profiles of CD138^+^ selected plasma cells from the BM of newly diagnosed MM patients (in the release used in this work (interim analysis 8, IA8), RNA-seq, together with clinical data, was available for 549 MM patients).

Datasets including MM cell lines gene expression profiling were retrieved from GEO database with the accession code GSE68379 and GSE6205. These data were normalized in Transcription analysis console (TAC, Thermo Scientific) software and result table processed through R Studio (R version: 3.5).

### Statistical analysis

Differences between means were analyzed by using GraphPad statistical package. The results were expressed as the mean ± SD of at least three different experiments, and the significance assessed by the two-tailed Student *t* test or Mann-Whitney test according to samples distribution. A *p* value of 0.05 or less was considered statistically significant. Overall survival (OS) and progression-free survival (PFS) analyses (Kaplan-Meier curves and log-rank test) have been performed by using SPSS statistical software on data retrieved by the CoMMpass database.

## Results

### NER genes are highly expressed in MM patients and correlate with prognosis

**Fig. 1:**
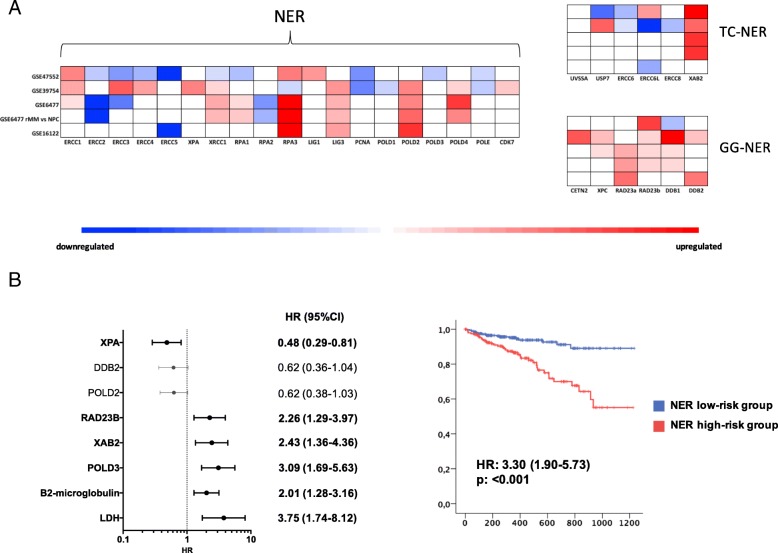
NER genes upregulation in MM cells and its prognostic correlation. **a** Meta-analysis of four different GEP datasets of MM comparing the expression levels of genes belonging to NER DNA-repair pathway in PCs from MM patients with PCs from healthy donors. **b** Shows a forest plot (on the left) reporting the results of the multivariate COX regression analysis performed on all NER-associated genes and on the 4 prognostic factors included in the R-ISS (B2-microglobulin, albumin, LDH, and adverse cytogenetic). A Kaplan-Meier curve (on the right) reports the results of the prognostic model built by dividing patients into NER “low” and “high” risk groups according to the previously selected genes expression. Importantly, patients in low-risk group experienced a significant hazard ratio reduction of about 70%

To establish the role of known DNA repair pathways in MM, a meta-analysis of all available gene expression profiling (GEP) data sets was performed by comparing the expression levels of genes belonging to each pathway (BER, NER, MMR, HR, c-NHEJ, a-NHEJ, FA) in primary cells from MM patients with normal PCs from healthy donors (gene lists are reported in Additional file 1: Table S1). As shown in Fig. 1a and Additional file 2: Figure S1A, upregulation of genes belonging to NER in MM cells, as compared to normal PCs, was found. Specifically, 7 out of 31 genes involved in the NER system were significantly deregulated in 4/5 datasets analyzed. Conversely, only 3/18, 0/10, 0/27, 0/15, 1/9, and 0/16 genes were deregulated in BER, MMR, HR, C-NHEJ, A-NHEJ, or FA respectively. Next, NER-associated genes were evaluated for their impact on MM patient prognosis by analyzing data from CoMMpass Trial (NCT0145429). To this aim, a multivariate COX regression analysis, including all NER-associated genes (31) and the 4 R-ISS variables, i.e., B_2_-microglobulin, albumin, LDH, and adverse cytogenetic [20] was performed. Four genes only (XPA, RAD23B, XAB2, and POLD3) showed independent predictive power (Fig. 1b left panel). Among them, a higher expression of RAD23B, XAB2, and POLD3 was associated with poor prognosis, whereas a higher expression of XPA was associated with better survival. Since Cox-regression model reported a similar relative contribution (positive or negative) for each variable, a score to each gene (low expression = 1, mid expression = 2, and high expression = 3) was assigned and then a prognostic risk score (RS) was built as follows: RAD23B + XAB2 + POLD3 − XPA. According to the median RS, patients were divided into NER “low” and “high” risk groups (Fig. 1b right panel). The prognostic model strongly associated with survival, with patients in low-risk group experiencing a significant hazard ratio reduction of about 70% (Fig. 1b right panel). Interestingly, the same analysis in all other DNA repair systems (Additional file 2: Figure S1B) did not reached results with the predictive value or NER-based score.

The finding that upregulation of different NER genes in MM PCs was correlated to poor prognosis suggested a major role for NER in MM pathobiology. On this basis, the efficacy of NER-targeting agents in this setting was investigated. Therefore, the anti-MM activity of trabectedin (Ecteinascidin 743), whose mechanism of action relies on NER system expression [21], was studied. Specifically, this drug binds to the minor groove of DNA and traps the NER machinery as it attempts to repair DNA, leading to the generation of lethal DNA double strand breaks. To our knowledge, the antitumor activity of trabectedin in MM has not been described so far.

### Trabectedin exerts potent anti-MM activity in vitro and in 3D models

**Fig. 2:**
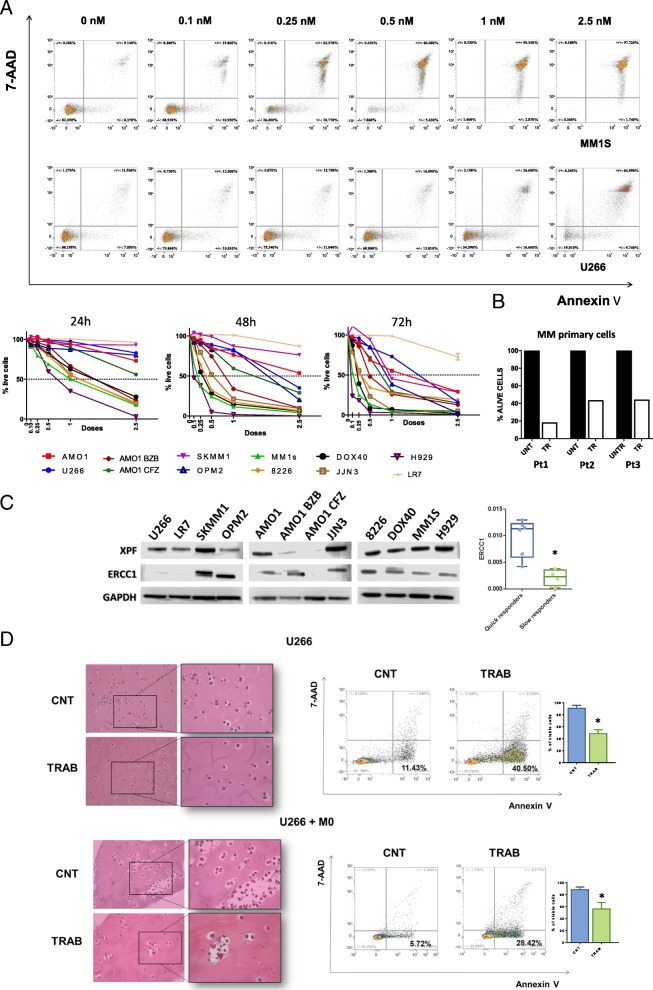
Activity of trabectedin in MM cells. **a** Representative dot plots of dose-dependent apoptosis increase in U266 and MM1S cells after dose escalation of trabectedin evaluated with annexin-V/7aad assay. In the lower left panel are represented the results of trabectedin activity on 12 MM cell lines at different time points. Each point represents the average result of at least 3 replicates. All cell lines (except for LR7) were very sensitive to trabectedin, with an IC50 at 72 h ranging from 0.5 to 2.5 Nm (%viable cells at the IC50 point significantly lower as compared with the control point, *p* < 0.01 for all cell lines). **b** Trabectedin activity on primary MM cells derived from 3 different patients, exposed to 2.5 nM of the drugs. After 24 h, we observed over 50% apoptosis evaluated with annexin-V/7aad assay. **c** Western blots reporting the expression of XPF and ERCC1 proteins in all the cell lines evaluated for sensibility to trabectedin treatment. On the right, evaluation of differences in protein expression after quantization, normalization and removal of outliers through ROUT test (*Q* = 1%). **d** A representative immunohistochemistry images of 3D model of U266 MM cells (in the absence or presence of macrophages) growth in matrigel-based spheroids, demonstrating the reduction of proliferation niches in trabectedin treated cells respect to control. The right dot plots showed the pro-apoptotic effects of trabectedin both in presence and in absence of macrophages (representative experiment of three) and the respective histograms showing the mean of three different experiments. *p < 0.05

The activity of trabectedin against MM cells was next investigated. Firstly, a panel of 12 MM cell lines, including several drug-resistant derived cells, was evaluated for sensitivity to increasing doses of trabectedin. Overall, all MM cell lines (except for LR7) were very sensitive to trabectedin exposure, with significant IC50 values for apoptosis at 72 h ranging from 0.5 to 2.5 nM (Fig. 2a). Moreover, the anti-MM activity of trabectedin was confirmed on primary cells from three different relapsed MM patients, by exposing cells to 2.5 nM of the drug. Importantly, also in this setting, over a significant 50% increase in apoptosis after 24 h (Fig. 2b and Additional file 3: Figure S2A) was observed (*p*: 0.02). Interestingly, we observed a dichotomic pattern of response after 24 h of treatment with trabectedin among all cell lines evaluated. Specifically, as reported in Fig. 2a, more than 50% of cells belonging to RPMI8226, DOX40, JJN3, H929, MM1S, and AMO-BZB cell lines were dead with 2.5 nM of trabectedin. We called these cells “quick responders,” and, as shown in Fig. 2c, by investigating the expression of several proteins belonging to the NER system, we observed that they expressed a significant higher protein level of ERCC1 as compared to “slow responders.” Indeed, the complex ERCC1/XPF is necessary for the formation of the trabectedin-induced DNA-DSB and ERCC1 expression has already been associated with response to trabectedin in cancer patients [22, 23]. As showed in Additional file 3: Figure S2B, none of the other evaluated proteins (XRCC1, DDB2, RPA32) exhibited a pattern associated with response to trabectedin. Along the same line, we investigated the expression of genes belonging to the NER pathway by interrogating two different publicly available datasets (GSE68379 and GSE6205) including several of the cell lines we used in our experiments [24, 25]. Unfortunately, while in both datasets, cells segregate in an unsupervised hierarchical clustering accordingly to their response to trabectedin (quick vs slow responders, Additional file 3: Figure S2C), we were unable to find significantly deregulated genes (a trend was observed for DDB1, CETN2, and POLD4; data not shown).

To strengthen our findings, trabectedin was further studied by a new relevant 3D model in which MM cells grow in matrigel-based spheroids. In this model, U266 cells form multicellular *niches* recapitulating the bone marrow structure of MM patients. The addition of trabectedin to Matrigel-spheroids impaired the development of MM *niches* (Fig. 2d upper panel). The 3D architecture mimics the protective effect of BM tissue structure on MM cells from drug-induced cytotoxicity and, indeed, a slight reduction of trabectedin activity in this model, as compared to 2D classic cultures of several MM cell lines (U266, Fig. 2d; MM1S and OPM2, Additional file 3: Figure S2D), was observed. However, this model does not fully recapitulate the BM microenvironment (BMM). To overcome this limitation, taking into account that macrophages infiltrate the BMM and protect MM cells from DNA-damaging [26, 27], Matrigel-spheroids in which MM cell lines (U266, MM1S, and OPM2) were co-cultured with healthy donors-derived monocytes were generated. As expected, after 72 h, monocytes/macrophages greatly improved the proliferative activity and viability of MM cells, and strongly promoted the generation of multi-cellular “microenvironmental” *niches*. Nevertheless, trabectedin overcame monocyte-dependent protective effects and significantly induced apoptosis in all MM cell lines tested (U266, MM1S, and OPM2) (Fig. 2d lower panel and Additional file 3: Figure S2D).

### Trabectedin modulates the transcriptome and induces cell cycle arrest, cellular stress, and DNA damage in MM cells

**Fig. 3:**
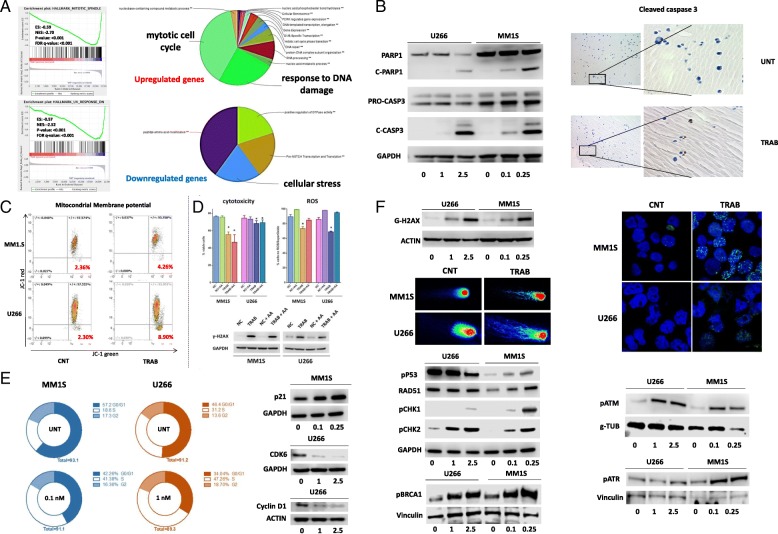
Trabectedin affects many cellular pathways. **a** On the upper left panel, the enrichment plots show significant gene-sets identified using GSEA software on U266 RNA. The green curve represents the enrichment score curve. On the right, the pie charts report different gene sets with up- or downregulation involved in trabectedin activity. **b** Western blot data showing levels of pro-apoptotic proteins c-PARP and c-caspase 3 after trabectedin treatment in U266 and MM1S cells. On the right, immunohistochemistry images showed brown foci of activated c-caspase 3 in trabectedin-treated U266 3D model compared to control. **c** Dot plots demonstrated modifications in mitochondrial membrane potential in MM1S and U266 cells trabectedin-treated respect to control. **d** Histograms representative of three independent experiments showed apoptosis induction and ROS production, after trabectedin treatment in U266 and MM1S cells, in the presence or absence of ascorbic acid (25uM) as antioxidant. In the lowest part, Western blots reporting the expression of gamma-H2AX in U266 and MM1S alone or in the presence of trabectedin and/or ascorbic acid. **e** On the left, a representation of the four phases of cell cycle, in two MM cell line (blue chart for MM1S and orange for U266), untreated or exposed to trabectedin. On the right, Western blot reported the expression of protein involved in cell cycle: p21 in MM1S and cyclin D1 and CDK4 in U266. **f** A representation of Western blot of g-H2ax expression in U266 and MM1S cells, in the upper panel. In addition, in single-cell gel electrophoresis (Comet) assay DNA strand breaks are identified through structures resembling comets, observed by fluorescence microscopy. The representative images showed the DNA damage in U266 and MM1S cell lines treated with 2.5 and 0.1 nM of trabectedin, respectively. Western blot images showed different proteins involved in DNA damage and belonging to DNA repair system. On the right top, immunofluorescence image showing green g-H2ax foci within MM1S and U266 nuclei exposed to 0.1 nM and 2.5 nM of trabectedin, respectively. *p < 0.05

To shed light on molecular mechanisms underlying the anti-MM activity of trabectedin, modifications in the gene expression profile of U266 cells, untreated or treated with 2.5 nM of trabectedin for 24 h (unsupervised hierarchical clustering is reported in Additional file 3: Figure S2E), were investigated by performing a GSEA [13]. By using both “hallmark” and “c2 curated” GSEA gene-sets, UV response/DDR and mitotic spindle formation (i.e., cell cycle progression) gene-sets were the most significantly modulated putative functions (Fig. 3a and Additional file 3: Figure S2F). Then, by taking advantage of the “ClueGO” and “CluePEDIA” apps in Cytoscape [14, 15], the main putative clusters of functions modulated by genes upregulated or downregulated by trabectedin were identified (Fig. 3a and Additional file 4: Figure S3A). Specifically, pathways associated with apoptosis, cell-cycle, DNA-damage, and cellular stress were the most significantly modulated. Additionally, among genes belonging to NER signaling, trabectedin significantly increased the expression of DDB2 (FC: 1.57), XPC (FC: 1.34), PCNA (FC: 1.7), and RPA2 (FC:1.45) (Additional file 4: Figure S3B; DDB2 upregulation in two cell lines was also confirmed by Western blot).

We then performed an in vitro validation of these bioinformatics findings.

It is well known that in other malignancies, trabectedin-induced apoptosis may occur in a P53-dependent or independent manner [28]. Accordingly, in our experimental setting, an increase of markers of late apoptosis, cleaved (c)-PARP and c-caspase3, in both P53-WT MM1S and P53-mutated U266 cell lines after 48 h treatment (Fig. 3b left panel) was observed. By immunohistochemistry, an increase of c-caspase 3-positive U266 cells from our 3D model after trabectedin treatment (Fig. 3b right panel) was further confirmed. Moreover, in U266 cells, a stronger reduction in the anti-apoptotic protein BCL-2 than in MM1S was detected (Additional file 5: Figure S4A). This effect was paralleled by a deepest alteration in mitochondrial membrane polarization (Fig. 3c). Additionally, in both cell lines, ROS and superoxide production significantly increased, confirming the predicted activation of stress response pathways (Fig. 3d and Additional file 5: Figure S4B). Then, to investigate whether DNA damage and apoptosis were correlated with ROS production, both U266 and MM1S cell lines were co-treated with trabectedin and ascorbic acid as antioxidant. Notably, despite a significant reduction in ROS levels, the antioxidant did not protect MM cells from trabectedin-induced apoptosis and DNA damage, evaluated as H2AX protein phosphorylation (γ-H2AX) (Fig. 3d), demonstrating both effects (DNA damage and apoptosis) to be independent from ROS production (representative dot plots of U266 and MM1S MM cells reporting trabectedin activity in term of apoptosis in presence or absence of trabectedin and/or ascorbic acid, were showed in Additional file 5: Figure S4B). Unfortunately, the exact mechanism of trabectedin-associated ROS production (already observed in other cellular systems [29]) is currently not fully understood. We may hypothesize that, at least in our settings, ROS are produced as a consequence of DNA double-strand breaks, DNA damage response activation, and mitochondrial membrane potential alteration.

Next, cell cycle alterations upon trabectedin were evaluated. In line with bioinformatics analysis, a trabectedin-dependent S-phase arrest with progressive decrease of cells in G0/G1 phases was observed (Fig. 3e left panel). The scenario depicted by these results was further empowered by an increase in the expression of the cell-cycle inhibitor p21^WAF1^ in MM1S, and by downregulation of Cyclin D1 and CDK6 in U266 (Fig. 3e right panel).

Lastly, the DNA-damaging activity of trabectedin was studied. As a surrogate marker of DSBs, the expression of γ-H2AX, a variant of histone 2A that is phosphorylated in the presence of DNA DSBs, was evaluated. Specifically, H2AX has a key role in DNA repair machinery and its phosphorylation represents the first step to recruit DNA repair proteins [30]. Additionally, g-H2AX is organized in foci that are associated to DNA fragmentation during apoptosis and DNA damage [31]. By Western blot, a strong increase of phosphorylated H2AX in the presence of trabectedin (results for U266 and MM1S cell lines are reported in Fig. 3f, and for OPM2 in Additional file 5: Figure S4C) was observed. These findings were empowered by demonstration of γ-H2AX foci within the nuclei of U266 and MM1S cells by confocal microscopy and by immunohistochemistry in the 3D model of MM (Fig. 3f and Additional file 5: Figure S4D). Furthermore, DNA fragmentation by a COMET assay demonstrated that trabectedin induces DNA damage and loss of chromatin organization in both U266 and MM1S (Fig. 3f). Accordingly, in both cell lines, trabectedin induced increased expression of proteins involved in DDR, including pATM and pATR, their downstream mediators pChk1 and pChk2, RAD51, pBRCA1, and pP53 (the latter only in MM1S) (Fig. 3f).

### Trabectedin reduces MM-macrophages-induced angiogenesis

**Fig. 4:**
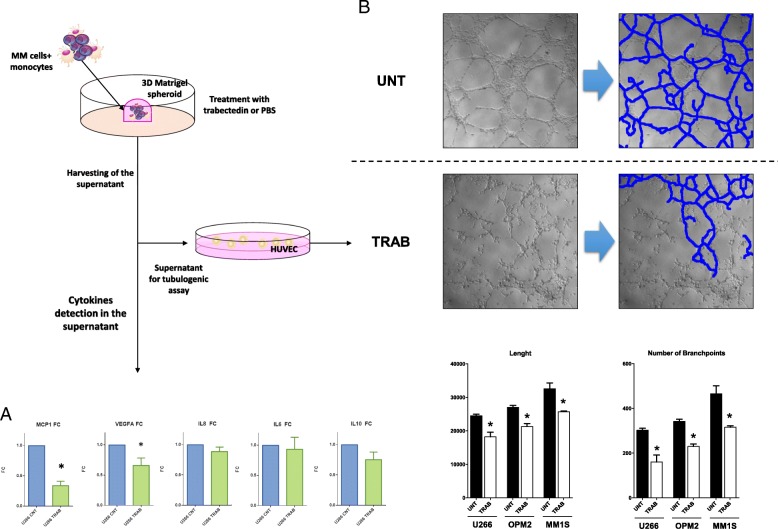
Trabectedin effects on angiogenesis. **a** Evaluation of cytokines production and secretion in the supernatant of 3D-spheroids of U266 and monocytes co-cultures treated with 2.5 nM of trabectedin. **b** Representative image from tubulogenic assay performed with supernatants of treated (2.5 nM of trabectedin) or untreated 3D-spheroids of U266-monocytes co-cultures. The image was analyzed through the “Pipeline 1.4” tool. In blue are reported the segments of neo-vessels/tubes. The histograms below show the tubulogenic potential, quantified by estimating the total tube length and the number of nodal branchpoints (a single pixel connected to 3 or more pixels), of the supernatant derived from 3D Matrigel-spheroids including monocytes co-cultured with 3 different MM cell lines. *p < 0.05

Due to the relevant role played by trabectedin as a microenvironment-modulating agent [32, 33], the concentration of a panel of cytokines/chemokines related with inflammation, chemotaxis, and angiogenesis in the supernatants of 3D-spheroids of monocytes-MM cells co-cultures (U266) alone or exposed to trabectedin was investigated. As shown in Fig. 4a, a significant reduction of MCP1, VEGF-A, and IL-10 (and a trend to reduced IL-8) in U266 and monocytes co-cultures was observed. Then, it was investigated if those modulations have any role in impairing MM-macrophages (M0)-induced angiogenesis. To verify our hypothesis, early passage HUVECs were cultured with conditioned medium obtained from 3D Matrigel-spheroids of MM (U266, OPM2, and MM1S) *plus* M0 in the presence or absence of trabectedin, after 72 h of culture. After 1 h, conditioned medium from untreated Matrigel-spheroids induced capillary-like structures to a higher extent as compared to the supernatant derived from trabectedin-treated co-cultures, as demonstrated by the significantly higher tube-like structure length and number of node branch points. This latter finding suggests anti-angiogenic activity for trabectedin in MM-associated BMM (Fig. 4b).

### Trabectedin increases the expression of NKG2D ligands in MM cells and induces NK activation

**Fig. 5:**
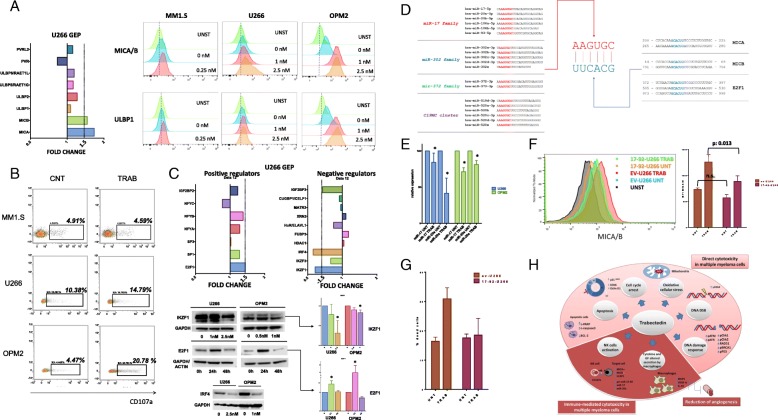
Trabectedin effects on MM recognition by innate immune system effectors. **a** On the left, expression levels of all known ligands of NK activating receptors derived from the results of the GEP of U266 treated with 2.5 nM of trabectedin. On the right, overlay histograms (scattered plots) underlying the modulation in MICA/B and ULBP1 expression in OPM2, U266, and MM1S, after trabectedin treatment. **b** Representative dot plot (of 3 different experiments) reporting the % of CD107a NK-92 after co-culture with different MM cells exposed to PBS or to 0.25 nM (MM1S), 1 nM (OPM-2), or 2.5 nM (U266) of trabectedin. **c** In the upper part, expression levels of negative or positive regulators of MICA/B derived from the results of the GEP of U266 treated with 2.5 nM of trabectedin. In the lower part, Western blot data showing protein expression of the previously identified MICA/B regulators: IKZF1, IRF4, and E2F1; the densitometric analysis of Western blot was performed on the triplicate blots for IKZF1 and E2F1 in both cell lines. **d** Sequences of miRNA families predicted to target at the same time the 3′UTR regions of MICA, MICB, and E2F1 (all upregulated after trabectedin treatment). These miRNAs present AAGUGC that interact with UUCACG motif of 3′UTR regions of previously reported genes. **e** Histograms reporting the expression of miR-17 and miR-20a in U266 and OPM2 cells untreated or exposed to 2.5 nM and 1 nM of trabectedin, respectively. **f** Overlay histogram reporting MICA/B expression in EV-U266 and 17-92-U266 cells alone or after treatment with 2.5 nM of trabectedin. On the right, histograms reporting the MFI of MICA/B of three different replicates. **g** Histograms showing apoptosis levels in EV-U266 as compared to 17-92-U266 after trabectedin treatment with 2.5 nM. **h** Cartoon representing the pleiotropic activity of trabectedin in MM, including direct cytotoxicity of trabectedin on MM cells, immune-modification of cytotoxicity on MM cells, and reduction of angiogenesis. *p < 0.05

Since DNA damage and cell stress have been found to increase NKG2D and DNAM-1 ligands expression on cancer cells, the role of trabectedin in MM cells susceptibility to NK-mediated cytotoxicity was investigated. Firstly, GEP analysis of known ligands (MICA, MICB, ULBP1-5, PVR, Nectin-2) of NK activating receptors (NKG2D and DNAM-1) demonstrated a significant upregulation of MICA and MICB genes (Fig. 5a). We confirmed such changes at protein level in several MM cell lines by flow cytometry: as shown in Additional file 5: Figure S4E, the highest increase in MICA/B was found in OPM2 cells. Additionally, trabectedin treatment significantly upregulated ULBP1 expression in the majority of cell lines analyzed (Fig. 5a and Additional file 5: Figure S4E). To functionally assess the relevance of these modulations, a degranulation assay was performed by measuring CD107a expression on natural killer cells (NK-92 CI) co-cultured with MM cells (U266, MM1S, OPM2, and RPMI8226) exposed to trabectedin. In line with the entity of ligands upregulation, the strongest significant degranulation on NK-92 cells co-cultured with trabectedin-treated OPM2 was observed, followed by RPMI8226 and U266 (Fig. 5b and Additional file 5: Figure S4F). Conversely, no NK activation was observed in the presence of trabectedin-treated MM1S where the NK ligands were not upregulated.

### Trabectedin activity is dependent from perturbation of transcription factors and microRNAs networks

To investigate the transcriptional mechanisms underlying trabectedin-induced MICA/B upregulation in MM cells, we explored in our GEP analysis of U266 cells the mRNA expression of known transcription factors involved in MICA/B transcriptional modulation [34]. Among MICA/B-positive regulators, only E2F1 was significantly upregulated, while, among negative regulators, IRF4 and IKZF1 were downregulated in MM cell lines treated with trabectedin as compared to control (Fig. 5c). Additionally, we further observed a trabectedin-dependent increase of the transcription of CRBN, CUL4, and DDB2 genes, all involved in the ubiquitination of IKZF1 protein (Additional file 5: Figure S4G).

E2F1, IRF4, and IKZF1 modulations were confirmed at protein level in U266 and OPM2 cell lines after 48-h exposure to trabectedin (Fig. 5c). Interestingly, while statistically significant, IKZF1 downregulation is far less pronounced in OPM2 than in U266 cell line, while E2F1 upregulation is stronger in OPM2. This could reflect a different weight of the two signaling in NKG2D ligands upregulation.

Moreover, to explore the post-transcriptional mechanisms correlated to trabectedin exposure in the regulation of MICA/B expression, we sought to examine whether trabectedin could regulate the expression of miRNAs. Indeed, by targeting the 3′ untranslated region (3′-UTR) of different mRNAs at the same time and, consequently, modulating their translation into proteins, miRNAs have a pivotal role in regulating almost all cellular function, including immune response. Indeed, increasing evidence reports on their capability of modulating, among others, the susceptibility of tumor cells to NK cell-mediated lysis [35–37]. On this basis, the miRNA target prediction tool mirDIP (http://ophid.utoronto.ca/mirDIP) has been used to identify miRNAs predicted to target, at the same time, MICA, MICB, and E2F1 mRNAs (which we previously demonstrated, in our experiments, to be upregulated after trabectedin treatment). As shown in Fig. 5d, all these miRNAs belong to well-characterized families, namely miR-17, miR-302, miR-372, and C19MC, all part of an oncogenic signaling network characterized by the presence of an AAGUGC sequence [38]. Interestingly, all these miRNAs modulate DNA damage response and cell cycle progression.

Taking into account the prominent role of miR-17-92 cluster in MM [10, 39–41] and the already reported capability of miR-17 family to modulate the expression of NKG2D ligands [42–44], the effects of trabectedin on the expression levels of miR-17 and miR-20a (the two miRNAs of the miR-17-92 cluster belonging to the miR-17 family) has been evaluated. As described in Fig. 5e and in Additional file 5: Figure S4H, trabectedin exposure significantly downregulated pri-miR-17-92, miR-17, and miR-20a in both U266 and OPM-2 cell lines. Importantly, as a control for the selectivity of trabectedin activity, two additional mature miRNAs of the miR-17-92 cluster (miR-19b and miR-92a), but not belonging to the miR-17 family, were not modulated after treatment (Additional file 5: Figure S4I).

Next, to assessed the role of these miRNAs in the regulation of NKG2DL expression, U266 cells were lentivirally transduced to generate a pri-MiR-17-92 overexpressing MM cell line (17-92-U266) (Additional file 5: Figure S4J, reports expression levels of mature miRNAs after transduction). Interestingly, 17-92-U266 cells demonstrated a lower (but not significantly) baseline expression of MICA/B respect to empty vector U266 cells (EV-U266), which levels increased significantly less after trabectedin treatment (Fig. 5f). We further observed an increased resistance to trabectedin-induced direct cytotoxicity in 17-92-U266 cells as compared to empty vector: as shown in Fig. 5g and in Additional file: Figure S4K, 17-92-U266 cells were significantly more resistant to trabectedin-induced apoptosis as compared to EV-U266 cells. Overall, these findings indicate a role for miR-17 family in trabectedin-dependent NKG2D ligands upregulation in MM cells.

## Discussion

In this work, through a gene expression dataset meta-analysis, we demonstrated that among DNA repair systems, NER is the most upregulated in MM and it is strongly associated with patients’ prognosis. On these bases, for the first time, we decided to evaluate the activity of NER-targeting agent trabectedin in this setting. Trabectedin has been granted approval for the treatment of advanced soft tissue sarcoma and for relapsed ovarian cancer, while several studies are ongoing to evaluate its activity in other malignancies [28]. In our experimental models, it exerts strong anti-myeloma activity on cell lines and primary cells at nanomolar concentrations, both in conventional 2D and in advanced 3D models. Interestingly, a quick response to trabectedin was associated to a high expression of ERCC1 protein, thus confirming its already known role in trabectedin mechanism of action [22, 23]. Additionally, trabectedin showed a pleiotropic activity in MM, which includes DNA DSBs generation, cell cycle arrest, exacerbation of cellular stress, reduction of angiogenesis, and immunomodulation (Fig. 5h). Regarding the latters, two different aspects have been of our interest. Several studies showed that trabectedin has significant effects on tumor microenvironment by impairing tumor-associated macrophages [32, 33]. Accordingly, in 3D Matrigel-spheroids, monocytes promoted MM cells viability and proliferation, which are strongly reduced by trabectedin. In the same setting, trabectedin modulated the pro-inflammatory cytokine/chemokine network by reducing MCP1, VEGF-A, and IL-10 which functionally translated into a decrease of pro-angiogenic potential. These effects are of particular relevance taking into account the role of inflammation and angiogenesis in MM [12, 45]. A relevant finding is, in our opinion, the triggering activity of trabectedin on the innate immune response against MM by increased expression of NKG2D ligands MICA/B and ULBP1. The relevance of NK response in MM pathogenesis has been deeply investigated [37, 46, 47], and in patients, a downregulation of surface expression of MICA on malignant plasma cells or a decline in NK-dependent immune-surveillance has been observed when MGUS progresses to symptomatic MM [48, 49]. Furthermore, the expression of killer cell inhibitory receptor (KIR) ligands, such as MHC class I, increases in advanced disease, impairing the balance between stimulatory and inhibitory signaling to promotion of NK inactivation [48]. Moreover, most of the anti-MM activity of immune-modulatory agents (IMiDs) has been attributed to their capability to induce proliferation and activation of NK cells [50]. Along this line, our study provides novel evidence that trabectedin-treated MM cells are more recognizable by innate immune effectors and that the drug strongly induces NK cell activation. These results are consistent with previous evidence reporting that low doses of doxorubicin or melphalan are able to increase NK activating receptors ligands with a mechanism dependent on DNA damage response [8].

In our work, we further investigated the regulatory network underlying trabectedin-mediated NKG2D ligands upregulation in MM. To this aim, the expression levels of all known MICA/B regulatory factors were evaluated by GEP and validated at protein level. Our results showed that trabectedin increased the expression level of the MICA/B-positive regulator E2F1 and reduced the expression of the negative regulators IRF4 and IKZF1. These findings are in line with recent reports where the phosphorylation of the kinases ATM/ATR, together with the production of ROS, induced the activation of E2F1 that, in turn, could promote MICA, MICB, and PVR transcription [51]. Additionally, the inhibition of IRF4 and IKZF1/3 by several drugs such as bromodomain and extra terminal domain inhibitors (BETi) and IMiDs has been found to induce MICA and PVR transcription [7, 52].

The mechanism through which trabectedin could affects E2F1, IRF4, and IKZF1 expression still remains elusive. Several reports in this field suggest the existence of possible networks between all these molecules that could account for the fine and reciprocal regulation we observed in this study [53–56]. Specifically, CUL4A, one of the four components (together with DDB1, CRBN, and DDB2) of a complex ubiquitination machinery responsible for lenalidomide-induced IKZF1 and IKZF3 degradation, has been demonstrated to be necessary for trabectedin activity [56]. Additionally, we observed an upregulation of three of the four genes of the ubiquitination machinery after trabectedin treatment. However, the strong activation of caspase 3 quickly induced by trabectedin treatment may account for the degradation of IKZF1 [57], with a mechanism that is independent from CRBN expression (differently from what observed with IMiDs). Both mechanisms could than contribute to IKZF1 downregulation that in turn inhibits IRF4 expression at transcriptional level [58], reducing the repressive regulation on MICA/B promoter.

On the other side, E2F1 has been demonstrated to be induced in response to several DNA-damaging agents, including UV radiation and a number of chemotherapeutic drugs [59]. This translates in an increase in protein stability and in some cases apoptosis [59]. Furthermore, taking into account that reprogramming the immune response requires rapid changes at both transcriptional and post-transcriptional level, we hypothesized a role for miRNAs in finely tuning this regulatory network. By using miRNA target prediction tools, miR-17 family has been identified as the most relevant in MM biology predicted to target at the same time MICA, MICB, and E2F1. We then confirmed that trabectedin downregulates miR-17 and miR-20a in MM cells and that miR-17-92 stable overexpression produced by a lentiviral construct reduces trabectedin-dependent upregulation of NKG2D ligands, making MM cells resistant to drug-induced apoptosis. The latter result appears to be in line with recent findings where miR-17-92 upregulation was found to be associated with resistance to trabectedin [60]. Additionally, the downregulation of miR-17 and miR-20a may further increase the upregulation of E2F1 [10], contributing to cell cycle arrest, and MICA/B surface expression. Thus, trabectedin could induce E2F1 upregulation through both DNA damage and miR-17 and miR-20a downregulation. However, the mechanism by which trabectedin reduces miR-17-92 transcription and impairs the miR-17-92/E2F1 auto-regulatory loop [61, 62] is currently under investigation.

## Conclusion

Altogether, our results demonstrated a potent and pleiotropic preclinical activity of trabectedin in multiple myeloma. Specifically, we here demonstrated an overexpression of NER genes in malignant MM plasma cells that strongly correlates with patients’ prognosis. Accordingly, we found that trabectedin exerts cytotoxicity on both MM cell lines (including drug-resistant derivatives) and primary MM patients derived cells at nanomolar concentrations, by inducing apoptosis, as confirmed by upregulation of caspase3 and downregulation of BCL-2, and cell cycle arrest increasing S-phase. Trabectedin also induces ROS production, with activation of stress response pathways, and DNA damage, enhancing the cytotoxic effect on MM cells. Importantly, trabectedin overcomes microenvironment-induced resistance, impairs MM-macrophages-mediated neo-angiogenesis, and induces NKG2D ligands upregulation enhancing NK-mediated killing. On the basis of these findings, trabectedin emerges as a new potential agent for the treatment of MM that deserves further translational and clinical investigation.

## Additional files

Additional file 1: **Table S1.** List of genes included in DNA repair systems. (XLSX 11 kb)

Additional file 2: **Figure S1.** **A** Meta-analysis of 4 different GEP datasets of MM comparing the expression levels of genes belonging to different DNA-repair pathways (BER, MMR, HR, c-NHEJ, a-NHEJ, FA) in PCs from MM patients with PCs from healthy donors. **B** For each panel: on the left, forest plot showing the results of the multivariate COX regression analysis performed on all genes included in the specific DNA repair system. On the right, Kaplan-Meyer curve report results of prognostic system in which patients were divided into “low” and “high” risk group, according to the expression of genes identified by previous multivariate analysis. (PDF 605 kb)

Additional file 3: **Figure S2.** **A** Dot plots reporting pro-apoptotic activity of trabectedin after 24 h treatment in primary myeloma cells from three different patients. On the right, histogram reporting the % of viable cells. **B** Western blot images of a panel of 12 MM cell lines representing proteins belonging to NER pathway, which not exhibited a pattern associated with response to trabectedin. **C** Expression of the genes belonging to the NER pathway obtained by interrogating 2 different publicly available datasets (GSE68379 and GSE6205) including several MM cell lines used in our in vitro experiments. Cell lines segregate, in an unsupervised hierarchical clustering, accordingly to their response to trabectedin. **D** Dot plots of apoptotic activity of trabectedin in OPM2 and MM1S in presence (right) or absence (left) of monocytes, treated with 1 nM and 0.1 nM of the trabectedin, respectively in 3D model. **E** Unsupervised hierarchical clustering demonstrating that both duplicates achieved comparable results. In green cluster: trabectedin treated U266; in yellow cluster: control U266. **F** Shows the first 9 results of the gene set enrichment analysis according to their ranking. Importantly, 5/9 gene-sets affected involves DNA damages. Additionally, GSEA correctly identified that the whole transcriptome modulation may be dependent upon trabectedin treatment. *: *p* < 0.05. (PDF 1280 kb)

Additional file 4: **Figure S3.** **A** GSEA results according to clueGO grouped by functions dependent on upregulated or downregulated genes. **B** Genes belonging to NER pathway resulted to be upregulated following trabectedin treatment in U266. Below, western blot to confirm DDB2 upregulation in 2 different cell lines. (PDF 974 kb)

Additional file 5: **Figure S4.** **A** Western blot showing expression levels of anti-apoptotic proteins BCL-2 and MCL-1 in U266 and MM1S treated with different doses of trabectedin. **B** Representative dot plot of apoptosis induction and ROS production in U266 and MM1S cells after trabectedin-treatment respect to control, in presence or absence of ascorbic acid. **C** Western blot reporting protein expression of cell-cycle and DNA-damage regulators (p21, p-Chk2, RAD51 and gH2AX) in OPM2 cell line, after trabectedin treatment. **D** Representative immunohistochemistry showing gamma-h2ax foci (in brown) in the nuclei of U266 cells growth in matrigel-based spheroids, after 2.5 nM trabectedin treatment. **E** Surface expression of MICA/B and ULBP1 in U266, OPM2, RPMI8226 and MM1S cells treated with different concentrations of trabectedin for 48 h. All results represent the mean of at least 3 different individual experiments. **F** Dot plots report CD107a surface expression on NK-92 CI co-cultured with RPMI8226 untreated and exposed to trabectedin for 48 h. In the lowest part, histogram reporting the mean of the CD107a expression on NK92 co-cultured with U266 and OPM2 in 3 different experiments. **G** Gene expression levels of CRBN, CUL4 and DDB2 genes, all involved in the ubiquitination of IKZF1 protein, extracted from our gene expression profiling performed on U266 after 24 exposure to trabectedin. **H** Expression of pri-miR-17-92 in U266 and OPM2 in both cell lines after trabectedin treatment (2.5 Nm for U266 and 1 nM for OPM2). **I** Expression of miR-19b and miR-92a in U266 and OPM2 in absence or presence of trabectedin. These miRNAs do not belong to miR-17 family and were used as control for trabectedin specific activity. **J** Expression levels of mature miRNAs belonging to miR-17-92 cluster in stably pri-miR-17-92 overexpressing U266. **K** Representative dot plot of apoptotic activity of trabectedin treatment (2 .5nM) in 17–92-U266 cells respect to EV-U266 cells. *: *p* < 0.05. (PDF 950 kb)

## Abbreviations

a-NHEJ: Alternative non-homologous and joining
BER: Base excision repair
BM: Bone marrow
c-NHEJ: Classical non-homologous and joining
CoMMpass: Relating Clinical Outcomes in MM to Personal Assessment of Genetic Profile
DDR: DNA damage response
DSBs: Double-strand breaks
EC: Expression console
ECL: Enhanced chemiluminescence
ES: Enrichment score
FBS: Fetal bovine serum
GEP: Gene expression profiling
GSEA: Gene set enrichment analysis
HD: Healthy donors
HR: Homologous recombination
IMiDs: Immune-modulatory drugs
MM: Multiple myeloma
MMP: Mitochondrial membrane potential
MMR: Mismatch repair
NER: Nucleotide excision repair
NES: Normalized enrichment score
NK: Natural killer
OS: Overall survival
P/S: Penicillin/streptomycin
PBMCs: Peripheral blood mononuclear cells
PCs: Plasma cells
PFS: Progression-free survival
PIs: Proteasome inhibitors
ROS: Reactive oxygen species
RT-PCR: Real-time polymerase chain reaction
tRNA: Total RNA
