# Supplementary material for: Trabectedin triggers direct and NK-mediated cytotoxicity in multiple myeloma
Source: J Hematol Oncol. 2019 Mar 21;12:32. doi: 10.1186/s13045-019-0714-9 (PMC6429746; doi:10.1186/s13045-019-0714-9)
Supplement: Supplementary file 2 — Figure S1. A Meta-analysis of 4 different GEP datasets of MM comparing the expression levels of genes belonging to different DNA-repair pathways (BER, MMR, HR, c-NHEJ, a-NHEJ, FA) in PCs from MM patients with PCs from healthy donors. B For each panel: on the left, forest plot showing the results of the multivariate COX regression analysis performed on all genes included in the specific DNA repair system. On the right, Kaplan-Meyer curve report results of prognostic system in which patients were divided into “low” and “high” risk group, according to the expression of genes identified by previous multivariate analysis. (PDF 605 kb) [file 13045_2019_714_MOESM2_ESM.pdf]

Figure S1

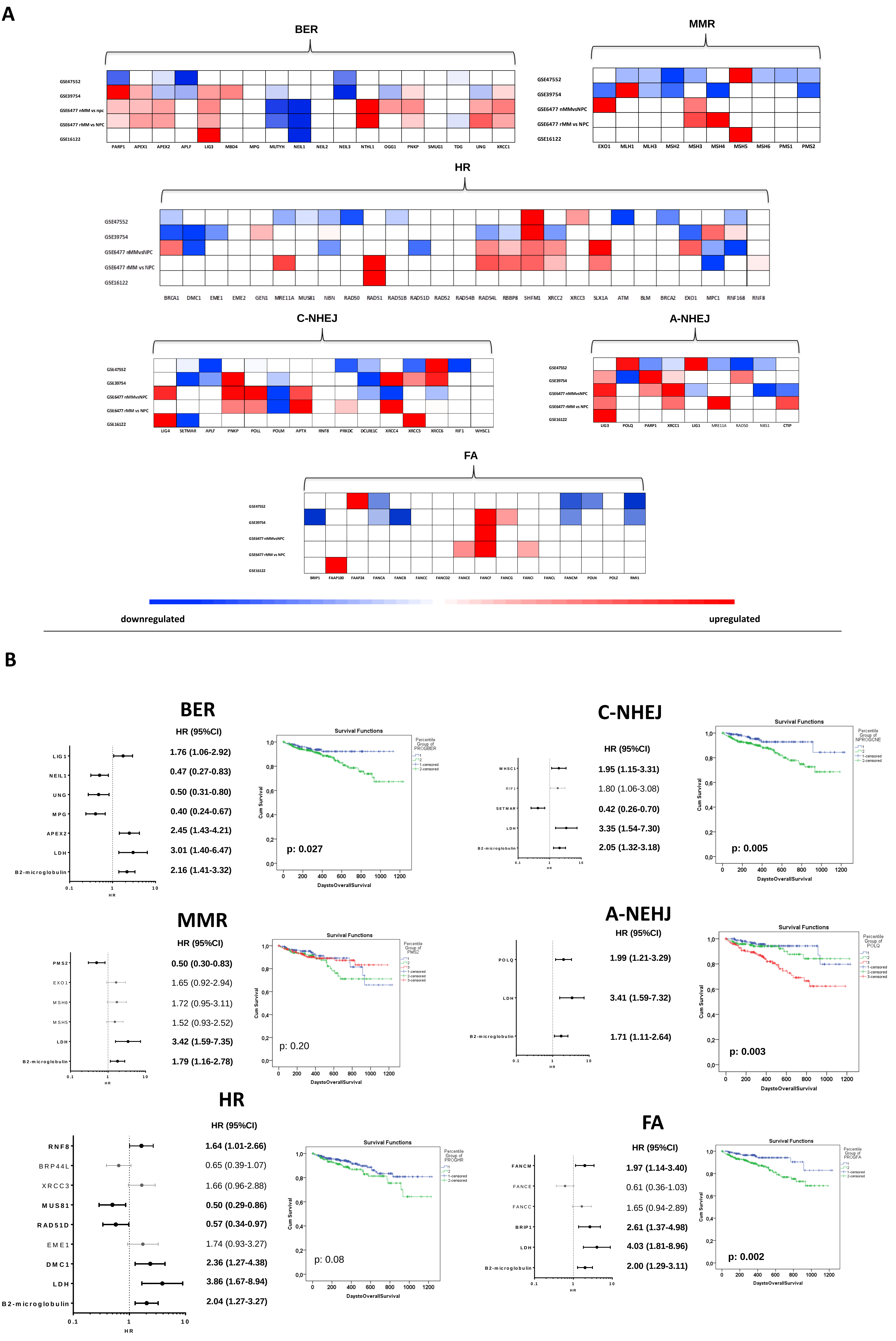
A
BER
GSE47552
GSE39754
GSE6477 nMM vs npc
GSE6477 rMM vs NPC
GSE16122
PARP1 APEX1 APEX2 APLF LIG3 MBD4 MPG MUTYH NEIL1 NEIL2 NEIL3 NTHL1 OGG1 PNKP SMUG1 TDG UNG XRCC1
MMR
GSE47552
GSE39754
GSE6477 nMMvsNPC
GSE6477 rMM vs NPC
GSE16122
EXO1 MLH1 MLH3 MSH2 MSH3 MSH4 MSH5 MSH6 PMS1 PMS2
HR
GSE47552
GSE39754
GSE6477 nMMvsNPC
GSE6477 rMM vs NPC
GSE16122
BRCA1 DMC1 EME1 EME2 GEN1 MRE11A MUS81 NBN RAD50 RAD51 RAD51B RAD51D RAD52 RAD54B RAD54L RBBP8 SHFM1 XRCC2 XRCC3 SLX1A ATM BLM BRCA2 EXO1 MPC1 RNF168 RNF8
C-NHEJ
GSE47552
GSE39754
GSE6477 nMMvsNPC
GSE6477 rMM vs NPC
GSE16122
LIG4 SETMAR APLF PNKP POLL POLM APTX RNF8 PRKDC DCLRE1C XRCC4 XRCC5 XRCC6 RIF1 WHSC1
A-NHEJ
GSE47552
GSE39754
GSE6477 nMMvsNPC
GSE6477 rMM vs NPC
GSE16122
LIG3 POLQ PARP1 XRCC1 LIG1 MRE11A RAD50 NBS1 CTIP
FA
GSE47552
GSE39754
GSE6477 nMMvsNPC
GSE6477 rMM vs NPC
GSE16122
BRIP1 FAAP100 FAAP24 FANCA FANCB FANCC FANCD2 FANCE FANCF FANCG FANCI FANCL FANCM POLN POLZ RMI1
downregulated
upregulated
B
BER
HR (95%CI)
LIG1 1.76 (1.06-2.92)
NEIL1 0.47 (0.27-0.83)
UNG 0.50 (0.31-0.80)
MPG 0.40 (0.24-0.67)
APEX2 2.45 (1.43-4.21)
LDH 3.01 (1.40-6.47)
B2-microglobulin 2.16 (1.41-3.32)
Survival Functions
Percentile Group of PROGBER
Cum Survival
DaystoOverallSurvival
p: 0.027
C-NHEJ
HR (95%CI)
WHSC1 1.95 (1.15-3.31)
RIF1 1.80 (1.06-3.08)
SETMAR 0.42 (0.26-0.70)
LDH 3.35 (1.54-7.30)
B2-microglobulin 2.05 (1.32-3.18)
Survival Functions
Percentile Group of NPROGCNE
p: 0.005
MMR
HR (95%CI)
PMS2 0.50 (0.30-0.83)
EXO1 1.65 (0.92-2.94)
MSH6 1.72 (0.95-3.11)
MSH5 1.52 (0.93-2.52)
LDH 3.42 (1.59-7.35)
B2-microglobulin 1.79 (1.16-2.78)
Survival Functions
Percentile Group of PMS2
p: 0.20
A-NEHJ
HR (95%CI)
POLQ 1.99 (1.21-3.29)
LDH 3.41 (1.59-7.32)
B2-microglobulin 1.71 (1.11-2.64)
Survival Functions
Percentile Group of POLQ
p: 0.003
HR
HR (95%CI)
RNF8 1.64 (1.01-2.66)
BRP44L 0.65 (0.39-1.07)
XRCC3 1.66 (0.96-2.88)
MUS81 0.50 (0.29-0.86)
RAD51D 0.57 (0.34-0.97)
EME1 1.74 (0.93-3.27)
DMC1 2.36 (1.27-4.38)
LDH 3.86 (1.67-8.94)
B2-microglobulin 2.04 (1.27-3.27)
Survival Functions
Percentile Group of PROGHR
p: 0.08
FA
HR (95%CI)
FANCM 1.97 (1.14-3.40)
FANCE 0.61 (0.36-1.03)
FANCC 1.65 (0.94-2.89)
BRIP1 2.61 (1.37-4.98)
LDH 4.03 (1.81-8.96)
B2-microglobulin 2.00 (1.29-3.11)
Survival Functions
Percentile Group of PROGFA
p: 0.002
