# Supplementary material for: Trabectedin triggers direct and NK-mediated cytotoxicity in multiple myeloma
Source: J Hematol Oncol. 2019 Mar 21;12:32. doi: 10.1186/s13045-019-0714-9 (PMC6429746; doi:10.1186/s13045-019-0714-9)

Figure S2

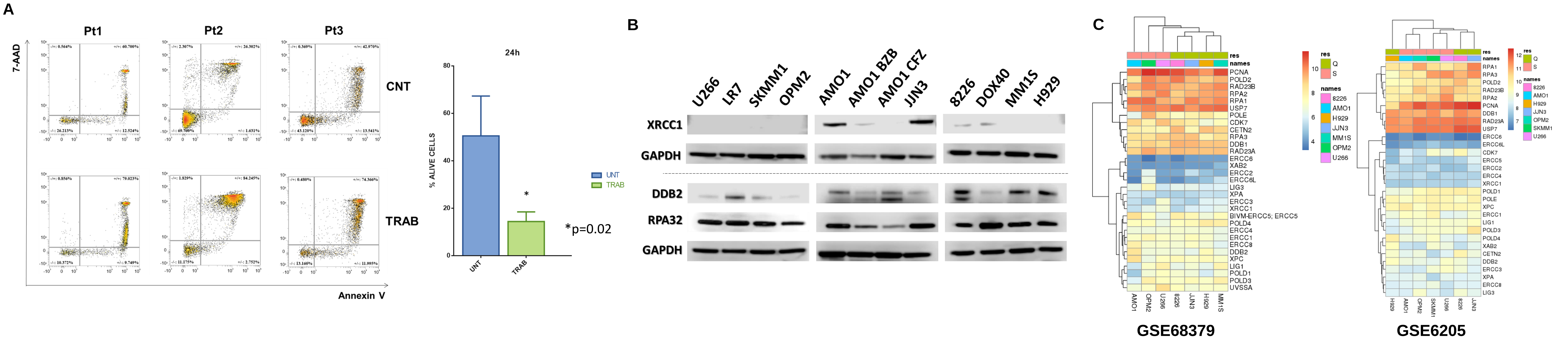

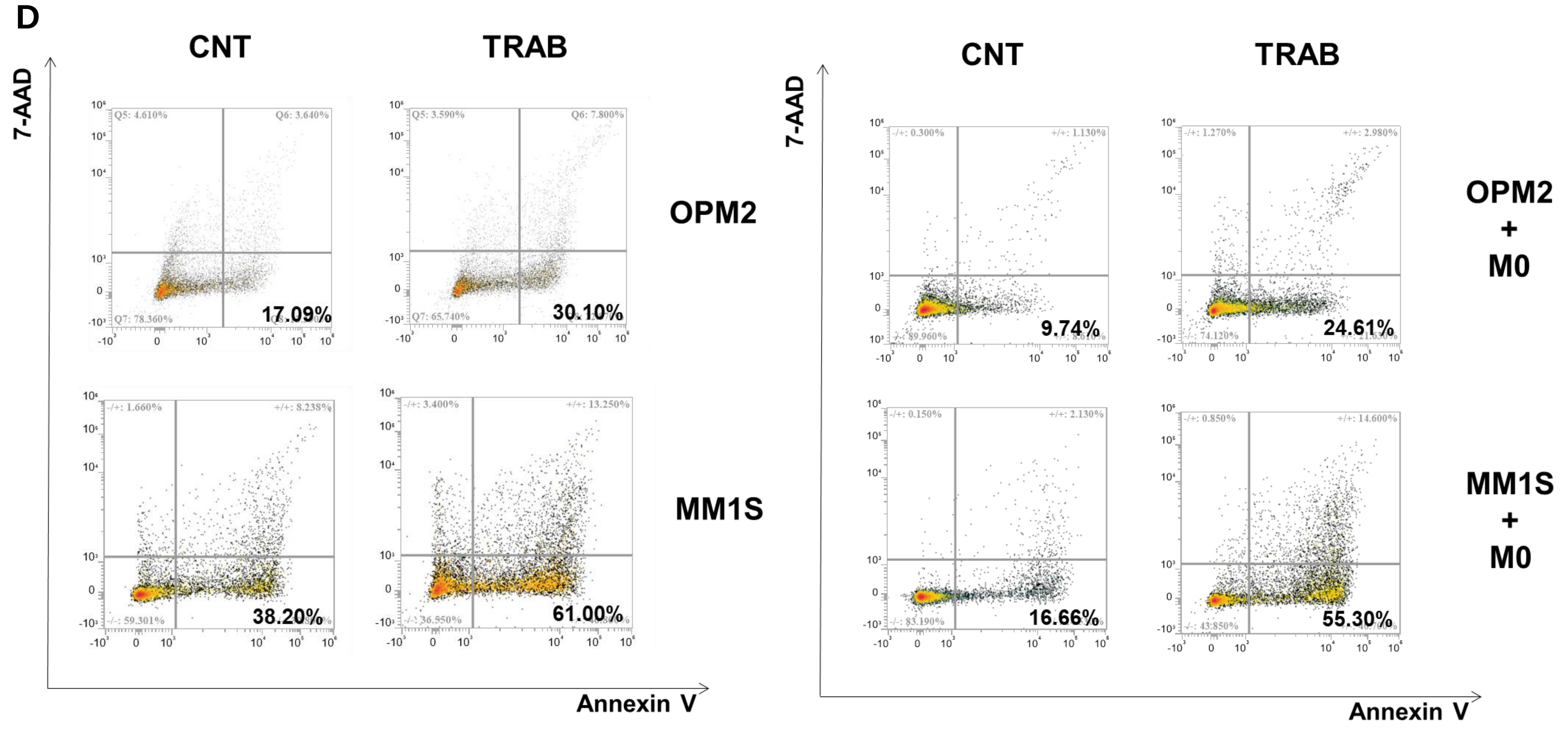

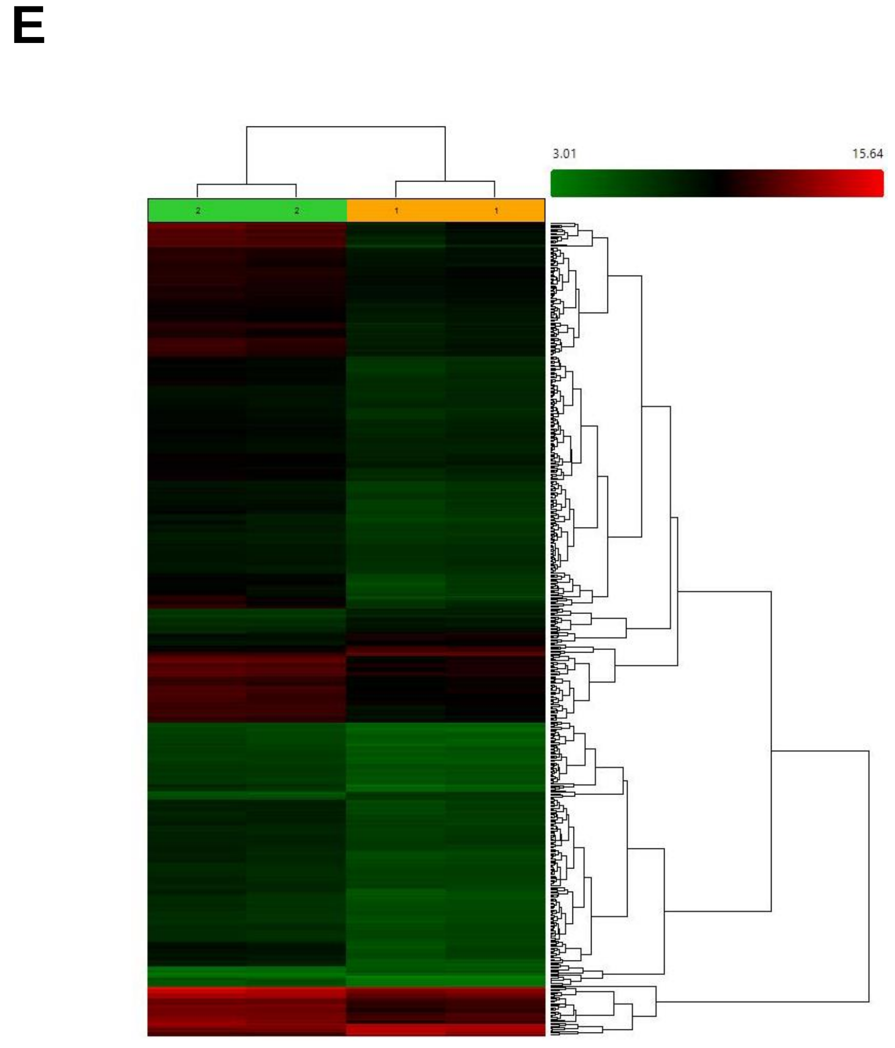

F

NAME	SIZE	ES	NES	NOM p-val	FDR q-val
**DACOSTA_UV_RESPONSE_VIA_ERCC3_COMMON_DN**	459	-0.75754225	-3.844.235	0.0	0.0
GABRIELY_MIR21_TARGETS	273	-0.67161334	-32.022.946	0.0	0.0
ZHENG_FOXP3_TARGETS_IN_THYMUS_UP	182	-0.6758235	-31.091.816	0.0	0.0
MARTINEZ_RESPONSE_TO_TRABECTEDIN	48	-0.859744	-30.599.437	0.0	0.0
IKEDA_MIR30_TARGETS_UP	112	-0.712233	-30.191.371	0.0	0.0
**DACOSTA_UV_RESPONSE_VIA_ERCC3_XPCS_DN**	83	-0.7537614	-30.155.766	0.0	0.0
**DAZARD_RESPONSE_TO_UV_NHEK_DN**	300	-0.6041593	-29.580.252	0.0	0.0
**GENTILE_UV_LOW_DOSE_DN**	64	-0.7594743	-29.258.807	0.0	0.0
**DACOSTA_UV_RESPONSE_VIA_ERCC3_TTD_DN**	78	-0.7146908	-29.214.928	0.0	0.0

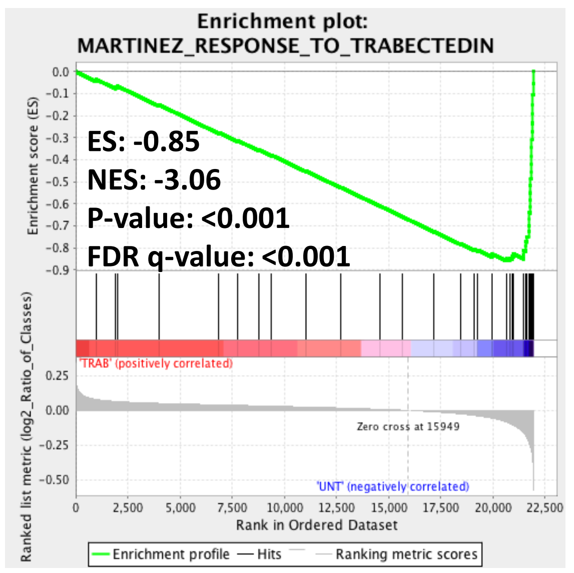

Supplement: Supplementary file 3 — Figure S2. A Dot plots reporting pro-apoptotic activity of trabectedin after 24 h treatment in primary myeloma cells from three different patients. On the right, histogram reporting the % of viable cells. B Western blot images of a panel of 12 MM cell lines representing proteins belonging to NER pathway, which not exhibited a pattern associated with response to trabectedin. C Expression of the genes belonging to the NER pathway obtained by interrogating 2 different publicly available datasets (GSE68379 and GSE6205) including several MM cell lines used in our in vitro experiments. Cell lines segregate, in an unsupervised hierarchical clustering, accordingly to their response to trabectedin. D Dot plots of apoptotic activity of trabectedin in OPM2 and MM1S in presence (right) or absence (left) of monocytes, treated with 1 nM and 0.1 nM of the trabectedin, respectively in 3D model. E Unsupervised hierarchical clustering demonstrating that both duplicates achieved comparable results. In green cluster: trabectedin treated U266; in yellow cluster: control U266. F Shows the first 9 results of the gene set enrichment analysis according to their ranking. Importantly, 5/9 gene-sets affected involves DNA damages. Additionally, GSEA correctly identified that the whole transcriptome modulation may be dependent upon trabectedin treatment. *: p < 0.05. (PDF 1280 kb) [file 13045_2019_714_MOESM3_ESM.pdf]
