# Supplementary figures and images for: Trabectedin triggers direct and NK-mediated cytotoxicity in multiple myeloma
Source: J Hematol Oncol. 2019 Mar 21;12:32. doi: 10.1186/s13045-019-0714-9 (PMC6429746; doi:10.1186/s13045-019-0714-9)

Figure S3

A  
Upregulated genes

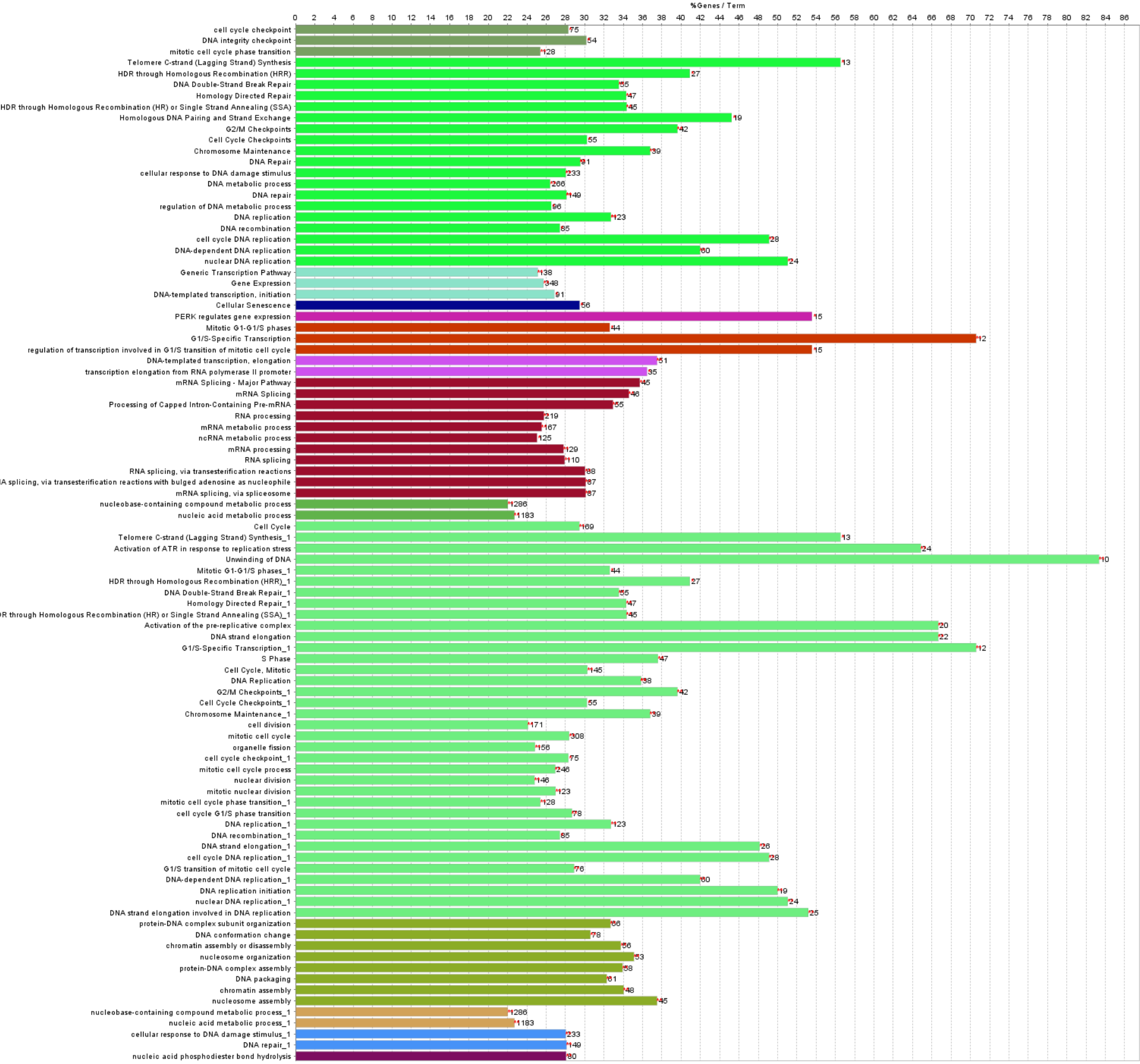

Downregulated genes

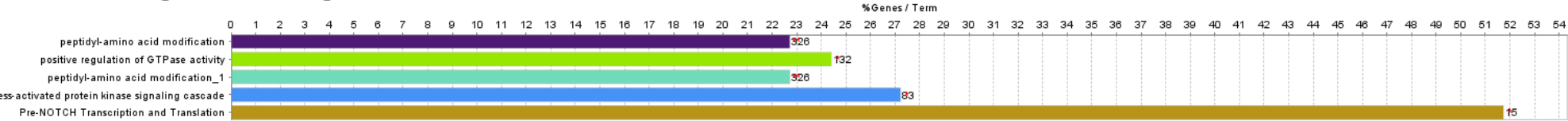

B

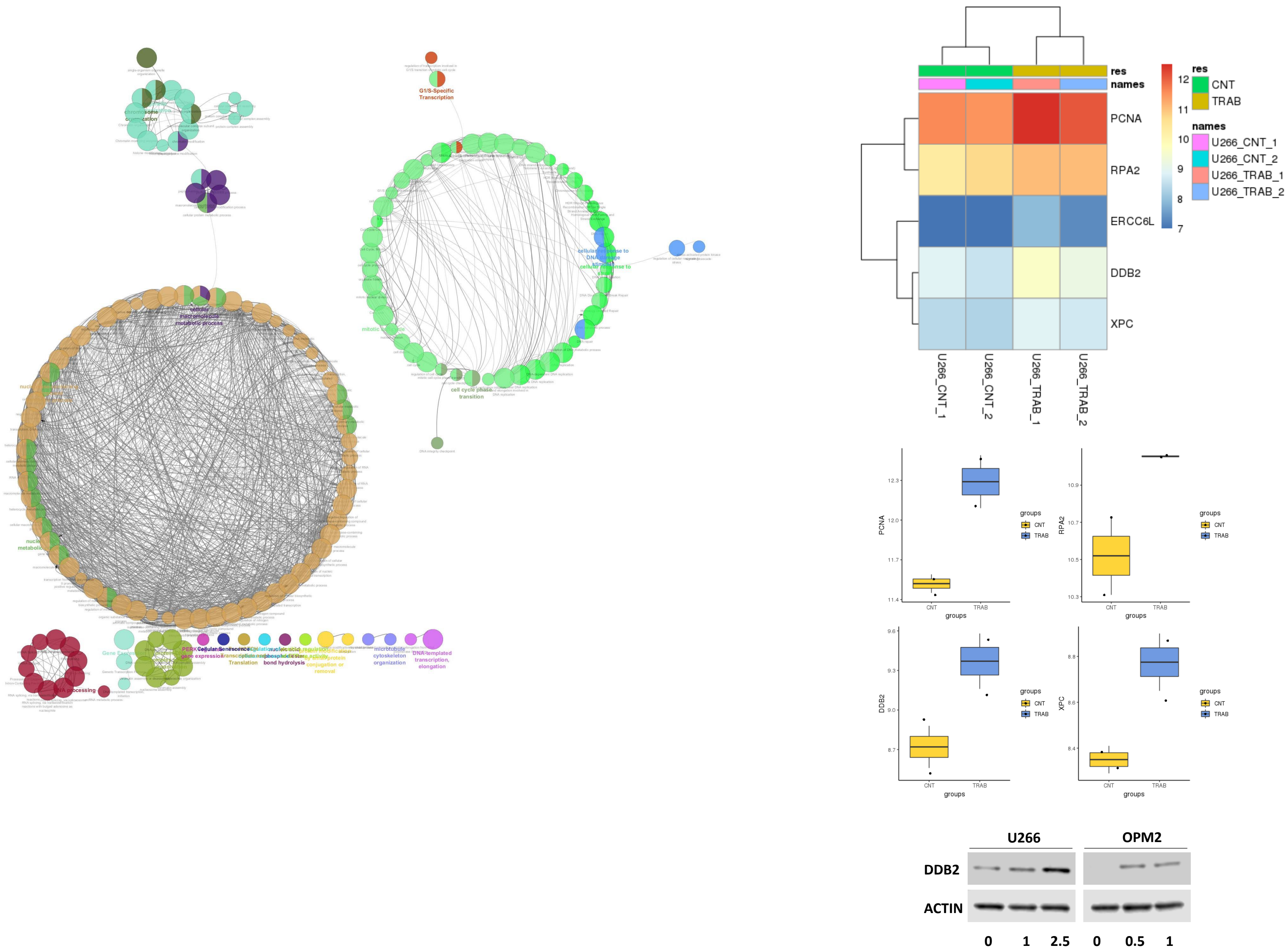

Supplement: Supplementary file 4 — Figure S3. A GSEA results according to clueGO grouped by functions dependent on upregulated or downregulated genes. B Genes belonging to NER pathway resulted to be upregulated following trabectedin treatment in U266. Below, western blot to confirm DDB2 upregulation in 2 different cell lines. (PDF 974 kb) [file 13045_2019_714_MOESM4_ESM.pdf]

Figure S4

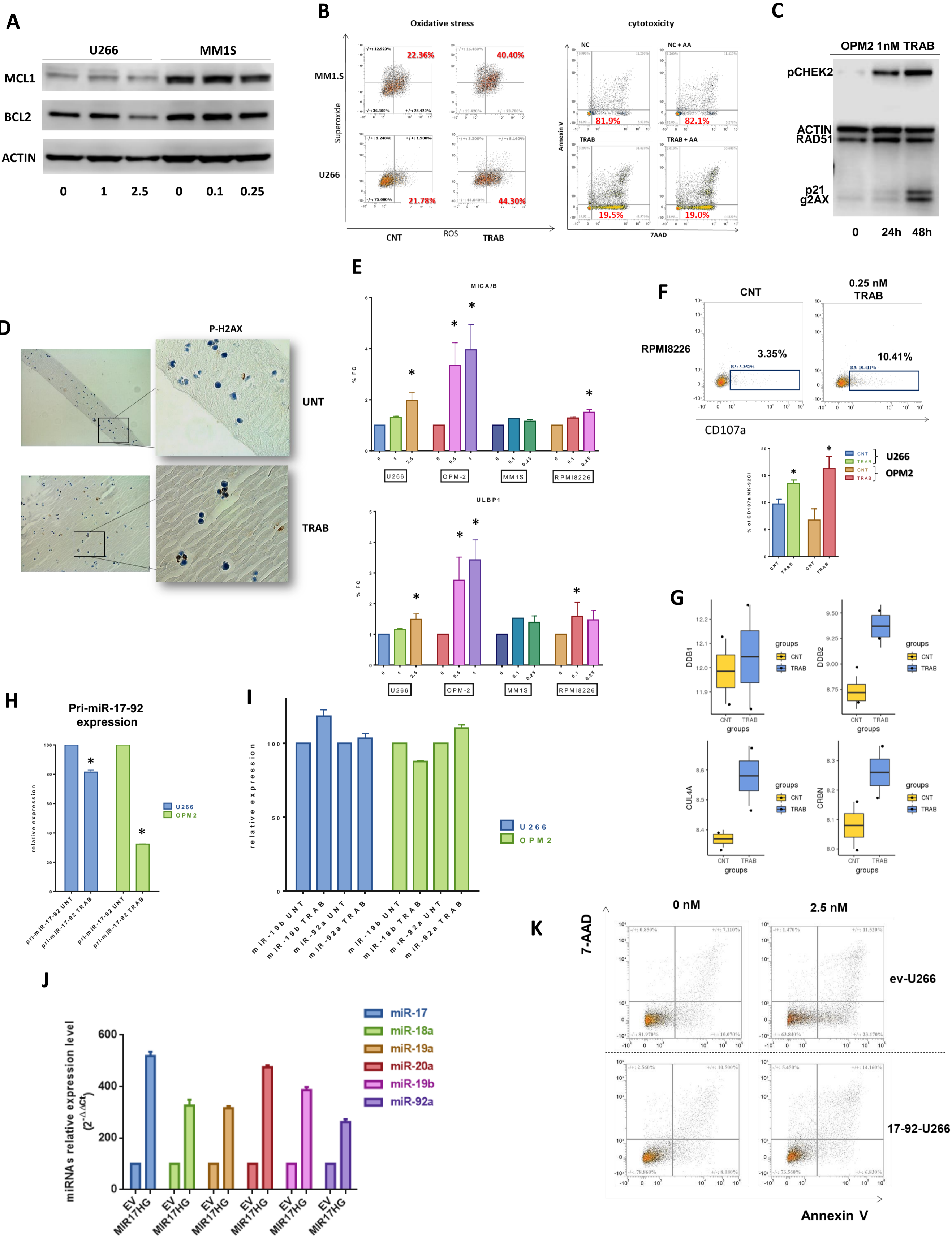

Supplement: Supplementary file 5 — Figure S4. A Western blot showing expression levels of anti-apoptotic proteins BCL-2 and MCL-1 in U266 and MM1S treated with different doses of trabectedin. B Representative dot plot of apoptosis induction and ROS production in U266 and MM1S cells after trabectedin-treatment respect to control, in presence or absence of ascorbic acid. C Western blot reporting protein expression of cell-cycle and DNA-damage regulators (p21, p-Chk2, RAD51 and gH2AX) in OPM2 cell line, after trabectedin treatment. D Representative immunohistochemistry showing gamma-h2ax foci (in brown) in the nuclei of U266 cells growth in matrigel-based spheroids, after 2.5 nM trabectedin treatment. E Surface expression of MICA/B and ULBP1 in U266, OPM2, RPMI8226 and MM1S cells treated with different concentrations of trabectedin for 48 h. All results represent the mean of at least 3 different individual experiments. F Dot plots report CD107a surface expression on NK-92 CI co-cultured with RPMI8226 untreated and exposed to trabectedin for 48 h. In the lowest part, histogram reporting the mean of the CD107a expression on NK92 co-cultured with U266 and OPM2 in 3 different experiments. G Gene expression levels of CRBN, CUL4 and DDB2 genes, all involved in the ubiquitination of IKZF1 protein, extracted from our gene expression profiling performed on U266 after 24 exposure to trabectedin. H Expression of pri-miR-17-92 in U266 and OPM2 in both cell lines after trabectedin treatment (2.5 Nm for U266 and 1 nM for OPM2). I Expression of miR-19b and miR-92a in U266 and OPM2 in absence or presence of trabectedin. These miRNAs do not belong to miR-17 family and were used as control for trabectedin specific activity. J Expression levels of mature miRNAs belonging to miR-17-92 cluster in stably pri-miR-17-92 overexpressing U266. K Representative dot plot of apoptotic activity of trabectedin treatment (2 .5nM) in 17–92-U266 cells respect to EV-U266 cells. *: p < 0.05. (PDF 950 kb) [file 13045_2019_714_MOESM5_ESM.pdf]
